# Overexpression of S100A4 is closely associated with the progression and prognosis of gastric cancer in young patients

**DOI:** 10.3892/ol.2013.1220

**Published:** 2013-02-28

**Authors:** HUA LI, ZIQUAN LIU, CHUANXIANG XU, YUNYUN CHEN, JIANWEI ZHANG, BO CUI, XUEWEI CHEN, GAIHONG AN, XIAOJUN SHE, HONGTAO LIU, ZIFENG JIANG, TIANHUI WANG

**Affiliations:** 1 Endoscopy Division, Tianjin Medical University Cancer Hospital and City Key Laboratory of Tianjin Cancer Center, Tianjin 300060;; 2 Institute of Health and Environmental Medicine, Tianjin 300050;; 3 Department of Physiology and Pathophysiology, Logistics College of Chinese People’s Armed Police Force, Tianjin 300162;; 4 Institute of Zoology, Chinese Academy of Sciences, Chaoyang, Beijing 100101, P.R. China

**Keywords:** S100A4, gastric cancer, young patients, progression, prognosis, immunohistochemistry

## Abstract

The aim of this study was to determine the correlation of S100A4 expression with the progression, prognosis and clinical pathology of gastric cancer (GC) in young pateints. A total of 85 tumor tissues with corresponding adjacent normal tissues and 62 non-metastatic lymph nodes (LNs) with corresponding metastatic LNs were obtained from young GC patients (<40 years old) who underwent surgery between January 2001 and December 2006. The expression of S100A4 was detected by RT-PCR and immunohistochemistry. Differences in the expression of S100A4 mRNA or protein were observed among the GC tissues, matched normal gastric mucosa, non-metastatic LNs and metastatic LNs. The expression of S100A4 mRNA and protein in GC tissues and metastatic LNs was significantly higher compared with that in the matched normal gastric mucosa and non-metastatic LNs, respectively (P<0.05). The overexpression of S100A4 was significantly associated with parameters involved in tumor progression and poor prognosis, including tumor size (P=0.017), Lauren classification (P=0.002), histological classification (P= 0.010), histological differentiation (P= 0.000), Borrmann classification (P=0.020), tumor-node-metastasis (TNM) stage (P=0.000), LN metastasis (P=0.000) and distant metastasis (P=0.024). Multivariate analysis suggested that patient age (P=0.035), tumor size (P=0.002), TNM stage (P=0.001) and S100A4 upregulation (P=0.000) were independent prognostic indicators for the disease. The overexpression of S100A4 in young GC patients is significantly associated with the clinicopathological characteristics. S100A4 may be used as a biomarker to predict the progression and poor prognosis of GC in young patients.

## Introduction

Gastric cancer (GC) is an extremely common disease worldwide and the second most common cause of cancer-related mortality (1). GC is considered to be a disease of the middle aged and elderly. However, a previous study revealed that 2 to 15% of patients with GC are younger than 40 years of age (defined as young GC patients), and that the relative proportion of young GC patients is higher than that of older GC patients (2). Despite the advances in chemotherapy and surgical techniques, the overall 5-year survival rate of GC patients in China remains low (3). At present, clinical staging and histopathological criteria are the only parameters used to stratify patients. However, the current staging classifications do not produce accurate predictions of patient outcomes. Molecular biomarkers may account for this diversity and several prognostic factors have been identified (4). However, none of these methods have been proven to be robust enough to be incorporated into routine practice.

S100A4 belongs to the S100 family, which is involved in the regulation of a wide range of intracellular and extracellular biological functions, including cell motility, differentiation and contractility, and is classified as a metastasis-related gene (5). A number of studies have suggested that S100A4 overexpression is correlated with poor clinical outcomes in various human cancers, such as bladder (6), colorectal (7), ovarian (8) and esophageal carcinoma (9). In the present study, immunohistochemistry (IHC) and RT-PCR were used to analyze the expression of S100A4 in 85 clinicopathologically characterized young GC patients.

## Patients and methods

### 

#### Patient population

A total of 85 young GC patients treated at Tianjin Medical University Cancer Hospital and Institute of China (Tianjin, China) between January 2001 and December 2006 were included in the study at the time of primary surgery for GC. The study was approved by the Ethics Committee of the hospital and informed consent was obtained from the patients. Patients with any of the following conditions were excluded from the present study: older than 40 years old, histology other than adenocarcinoma, preoperative chemoradiotherapy, mortality not caused by cancer and unknown stage of disease. The study population included 85 patients in tumor-node-metastasis (TNM) stages I–IV who had undergone surgery. Paraffin sections of tissues from these patients were prepared. The presence or absence of distant metastases was determined through radiological examination. Primary tumor sections were re-evaluated by an experienced pathologist who was blinded to the patients’ survival or other clinical variables to ensure consistent staging and grading. Representative sections for each tumor were identified and prepared for subsequent IHC analysis.

#### IHC and scoring

IHC staining was performed based on a previously described method (10) to identify changes in the protein expression of the primary GC, metastatic LN and their normal counterpart tissues. In brief, the slides were placed in an oven at 60°C for 2 h, deparaffinized with xylene and then rehydrated. The sections were submerged in an EDTA antigenic retrieval buffer, placed in a microwave for antigen retrieval, treated with 3% hydrogen peroxide in methanol to quench endogenous peroxidase activity and then incubated with 1% bovine serum albumin to block non-specific binding. The sections were incubated with mouse anti-S100A4 (Santa Cruz Biotechnology, Santa Cruz, CA, USA) overnight at 4°C. Normal goat serum was used as the negative control. The tissue sections were washed, treated with secondary antibody, counterstained with hematoxylin, dehydrated and then mounted.

S100A4 was stained yellow-brown in the cytoplasm and nucleus. The degree of immunostaining was reviewed and scored independently by two observers based on the staining intensity and percentage of positive cells. The intensity grading scale was according to the following criteria: 0 (no staining), 1 (weak staining, light yellow), 2 (moderate staining yellow-brown) and 3 (strong staining, brown). Moderate and strong staining indicated tumors with high S100A4 expression, while no and weak staining indicated low S100A4 expression.

#### RT-PCR

Total RNA was extracted from the primary GC, metastatic LN and their normal counterpart tissues using TRIzol reagent (Takara Biotech, Dalian Co., Ltd., Otsu, Japan). Reverse transcriptase reactions were performed using 1 mg of total RNA and PrimeScript™ RT reagent kit (Takara Biotech) followed by PCR amplification (TC-412 thermal cycler; Techne, Stone, Staffordshire, UK) with specific primers. PCR amplification was performed as follows: 30 cycles of 94°C for 5 min, 58°C for 30 sec and 72°C for 30 sec, with a final extension of 72°C for 10 min. After the PCR products were electrophoresed on 1.5% agarose gels, they were arrayed using a Bio-Rad scanner system (Hercules, CA, USA) and analyzed with Quantity One software. The specific primers for the S100A4 gene were S100A4-sense (5′-GATGTGATGGTGTCCAccTT-3′) and S100A4-antisense (5′-ATTTCTTCCTGGGCTGCTTA-3′) whose target was a 277-bp fragment. Primer pairs specific to the β-actin gene (β-actin-sense, 5′-CCAGATCAtGTTTGAGACCT-3′; β-actin-antisense, 5′-TTGAAGGTAGTTTCGTGGAT-3′; PCR product, 480 bp) served as the internal standard.

#### Postoperative follow-up

Following surgery, each patient was scheduled for a follow-up examination every 4 months in the first year, semi-annually in the second year and annually thereafter. More frequent examinations were scheduled if clinically indicated. The cause of mortality was registered and classified as mortality due to GC, other causes or unknown causes. For overall survival, the median follow-up of the surviving patients was 15 months (range, 2–81 months).

#### Statistical analysis

The associations between S100A4 staining and clinicopathological variables were tested using the two-tailed Fisher’s exact test or linear-by-linear association Chi-squared test. The Kaplan-Meier method was used to calculate the survival functions and differences were assessed with the log-rank test. Multivariate analysis was performed using the Cox proportional hazards regression model. Overall and disease-specific survival were determined from the time of surgery until mortality. P<0.05 was considered to indicate statistically significant differences. All reported P-values were two-sided and all analyses were performed using the Statistical Package for Social Sciences, version 19.0, for Windows (SPSS Inc., Chicago, IL, USA).

## Results

### Expression of S100A4

#### Expression of S100A4 in the gastric mucosa

**Figure 1:**
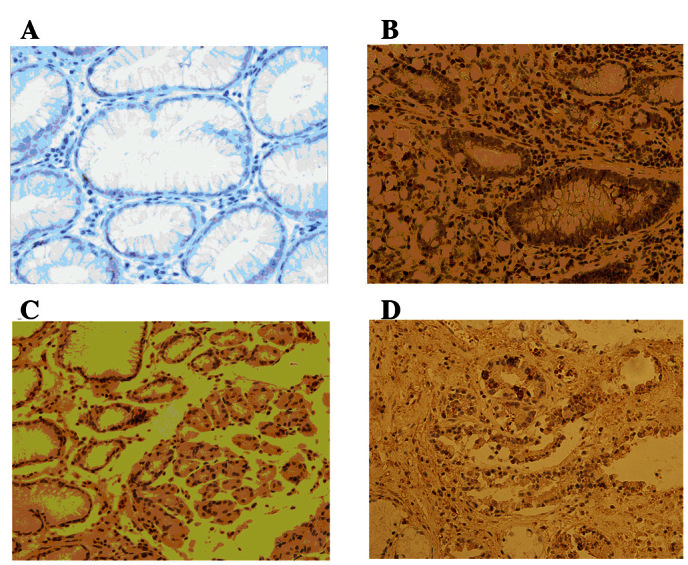
Expression of S100A4 in the gastric mucosa (magnification, ×400). (A) Normal gastric mucosa. (B) Gastric mucinous adenocarcinoma. (C) Poorly differentiated gastric adenocarcinoma. (D) Villous gastric adenocarcinoma.

**Figure 2:**
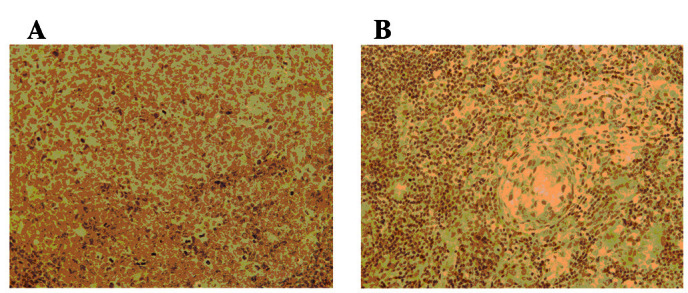
Expression of lymph node (magnification, ×400). (A) Non-metastatic lymph node, S100A4^−^. (B) Metastatic lymph node, S100A4^+^.

**Figure 3:**
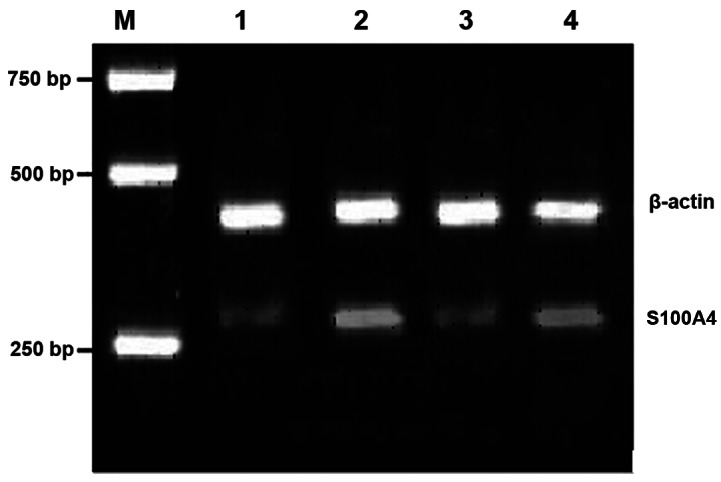
RT-PCR analysis of β-actin/S100A4 mRNA level in various tissues. M, DNA marker; 1, RT-PCR products from normal counterpart gastric tissues; 2, RT-PCR products of primary gastric cancer tissues; 3, RT-PCR products from normal lymph node tissues; 4, RT-PCR products from positive lymph node tissues. RT-PCR, reverse transcription-polymerase chain reaction.

**Figure 4:**
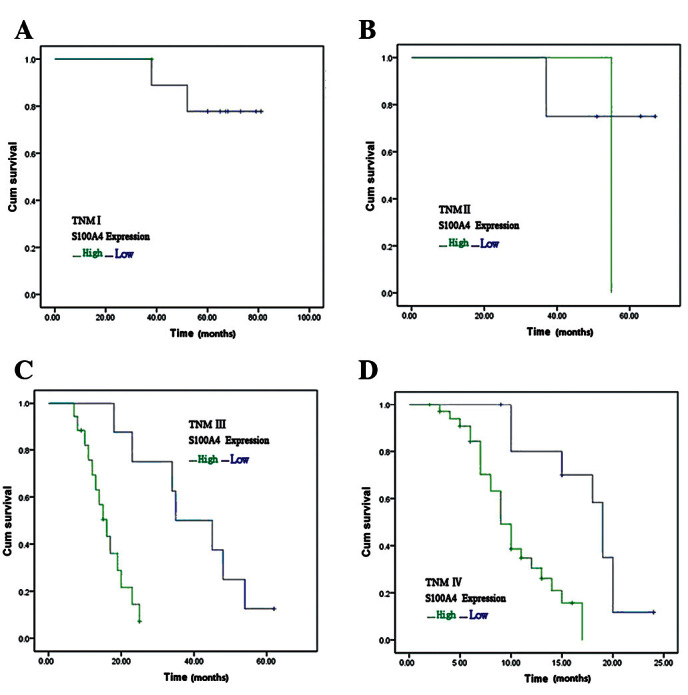
Kaplan-Meier curves with univariate analysis (log rank) for patients with low S100A4 expression vs. high S100A4 expression. (A) Stage I tumor. (B) Stage II tumor. (C) Stage III tumor. (D) Stage IV tumor. TNM, tumor-node-metastasis.

S100A4 protein was detected in 18 (21.18%) out of the 85 non-tumor mucosal samples obtained from young patients. The majority of the samples expressed the protein at a low level. The expression was negligible or negative in the normal gastric mucosa but moderate to strong in lymphocytes and smooth muscles cells. Among the 85 GC tissue specimens from young patients screened for S100A4 protein expression, 53 (62.35%) exhibited S100A4 overexpression, in which immunostaining was observed in the cytoplasm or the nucleus of the tumor cells (Figs. 1–4). Statistically, S100A4 overexpression was closely associated with the tumor size (P=0.017), Lauren classification (P=0.002), histological classification (P=0.010), histological differentiation (P=0.000), Borrmann classification (P=0.020), TNM stage (P=0.000), LN metastasis (P=0.000) and distant metastasis (P=0.024). However, no significant correlation was observed between S100A4 overexpression and other clinicopathological parameters, including age, gender, tumor location, surgery type and *Heliobacter pylori* infection (Table I).

#### Expression of S100A4 in the LN

The S100A4 protein was present in non-metastatic LNs at low levels (Fig. 2). The S100A4 protein level in the metastatic LNs was significantly higher than that of the non-metastatic LNs. S100A4 overexpression was closely correlated with LN staging (P=0.000; Table II). No significant difference in S100A4 expression was observed between the GC tissues and metastatic LNs (P=0.896).

#### Expression of S100A4 mRNA

S100A4 expression was detected using IHC. The relative expression level of β-actin/S100A4 in the primary GC (0.4493±0.0453) was higher than that in the normal tissues (0.1145±0.1000). In addition, the relative expression level of the metastatic LNs (0.5491±0.0197) was higher than that in the normal LN tissues (0.1558±0.0318). The difference between each group was statistically significant (P<0.05; Fig. 3).

#### Correlation between S100A4 expression and patient prognosis

In stage I–IV tumors, the 1- or 3-year survival rate of the patients with high S100A4 expression was significantly lower than that in the patients with low S100A4 expression (P<0.05; Fig. 4).

#### Multivariate analysis of clinicopathological parameters and prognosis

The factors with possible prognostic effects on young GC patients were analyzed by Cox regression. The results showed that patient age (P=0.035), tumor size (P=0.002), TNM stage (P=0.001) and S100A4 upregulation (P=0.000) were independent prognostic indicators for the disease (Table III).

## Discussion

A number of studies on the clinicopathological and molecular biological features of GC in the elderly have been performed. However, only a few studies with a small sample size have analyzed GC in young patients (11). Previous studies have associated S100A4 protein expression with survival in several tumor types, including bladder (6), colorectal (7), ovarian (8) and esophageal carcinoma (9). S100A4 protein expression has also been revealed to have prognostic significance in GC (12–14). In a study of 436 cases, Wang *et al*(12) demonstrated that IHC staining for S100A4 is associated with LN metastasis and poor prognosis in GC patients. The present study demonstrates the prognostic significance of S100A4 overexpression in the tumor cells of young GC patients for the first time. Multivariate analysis showed that S100A4 overexpression was associated with patient outcome. The observed results may translate into clinically important differences.

In certain studies (11,15), a female predominance was observed among young GC patients. However, a higher proportion of male patients has been noted in elderly GC patients (16).

In the present study, the female-to-male ratio was 1.5:1 in the young GC patients. The reason for the higher number of female patients is not yet known. In the present study, no significant difference in S100A4 expression was observed between the male and female patients (P>0.05).

Infection caused by *H. pylori* is considered to be an important epidemiological risk factor for GC patients of all ages (17,18). Moreover, certain epidemiological data have revealed an association between *H. pylori* infection and an increased risk of GC presenting at a young age (19,20). However, in the present study, no significant difference in S100A4 expression was observed in the patients with and without *H. pylori* infection. In addition, *H. pylori* positivity was infrequently observed among the young GC patients.

Chung *et al*(15) reported that the incidence of primary lesions in the upper third of the stomach is higher in young patients than in elderly patients. In the present study, the same results were reported, although no significant difference was observed.

Similar to the results of previous studies (11,15), the histology in young patients was observed to be more poorly differentiated.

Gastrectomy in combination with lymphadenectomy is the only potentially curative treatment for localized gastric carcinomas, and curative resection offers the only chance of long-term survival. However, radical resection is not common in young GC patients due to clinicopathological characteristics. Numerous studies have reported that the curative resection rate in young patients with GC is low. In the present study, the type of surgery showed no significant effect on S100A4 expression.

In the present study, quantitative RT-PCR revealed the presence of S100A4 mRNA in non-tumor mucosal samples from young patients. The non-metastatic LNs expressed S100A4 mRNA at low levels. The levels of S100A4 mRNA in the GC tissues and metastatic LNs were significantly higher compared with those in the matched normal gastric mucosa and non-metastatic LNs, respectively. Similarly, S100A4 expression was also detected using IHC. IHC showed that the S100A4 protein was associated with GC cells and other non-parenchymal cell types including lymphocytes. The proportion of specimens with epithelial cells stained for S100A4 increased between the GC tissues and metastatic LNs. However, no significant difference was observed. By contrast, the proportion of lymphocytes stained for S100A4 remained at a consistently high level in the normal gastric tissues, GC tissues and metastatic LNs. Significant differences in the expression levels of the S100A4 protein were observed among the TNM stages of GC, as well as between metastatic GC and non-metastatic GC. The results of the present study are consistent with the results of previous studies (12–14), in which S100A4 expression detected via IHC was increased in malignant relative to non-malignant GC specimens. Moreover, the deeper and more invasive regions of the specimens in these studies were enhanced in immunohistochemically detectable S100A4. This suggests that high levels of S100A4 protein are associated with the invading regions of the tumors. S100A4 overexpression was more frequently observed in the metastatic LNs than in the primary GC tissues from young patients. However, no significant difference was observed.

Previous studies reported that S100A4 overexpression is closely correlated with several factors for GC aggressiveness, such as LN metastasis, distant metastasis and TNM stage (12–14). In the present study, similar results were observed in the young GC patients. These data further support a significant correlation between S100A4 overexpression and GC progression, indicating the putative role of S100A4 in tumor cell aggressiveness.

In conclusion, S100A4 overexpression is an independent predictor of adverse prognosis in young GC patients. Thus, it may be used as a biomarker in future clinical studies.

## Figures and Tables

**Table I:** Association between S100A4 expression and clinicopathological characteristics.

	S100A4			
Characteristics	Low	High	χ^2^	P-value
Age (years)			0.134	0.935
<30	6	11		
31–35	9	16		
36–40	17	26		
Gender			1.011	0.315
Male	15	19		
Female	17	34		
Tumor location			0.894	0.640
Proximal	5	11		
Middle	10	12		
Distal	17	30		
Tumor size (cm)			5.647	0.017
<5	18	16		
≥5	14	37		
Surgery			3.348	0.341
Distal subtotal gastrectomy	17	28		
Proximal subtotal gastrectomy	5	9		
Total gastrectomy	9	9		
Other	1	7		
Lauren classification			9.465	0.002
Intestinal	17	11		
Diffuse	15	42		
Histological classification			13.326	0.010
Tubular adenocarcinoma	14	10		
Papillary adenocarcinoma	5	3		
Mucinous adenocarcinoma	7	11		
Poorly differentiated adenocarcinoma	6	25		
Other	0	4		
Histological differentiation			18.501	0.000
Well	8	1		
Moderately	7	3		
Poorly	17	49		
Borrmann classification (type)			9.890	0.020
I	6	1		
II	11	13		
III	11	27		
IV	4	12		
Tumor-node-metastasis stage			19.178	0.000
I	9	1		
II	4	1		
III	8	17		
IV	11	34		
Lymph node metastasis			27.400	0.000
N0	12	2		
N1	7	4		
N2	10	19		
N3	3	28		
Distant metastasis			5.070	0.024
M0	31	46		
M1	0	8		
*H. pylori*			3.108	0.078
(+)	13	12		
(−)	19	41		

**Table II:** Association between S100A4 expression and LN metastasis.

	S100A4			
Characteristics	Low	High	χ^2^	P-value
LN metastasis			14.409	0.000
Negative	45	17		
Positive	24	38		
No. of LN metastases			14.409	0.000
1–6	13	2		
7–15	7	15		
>15	4	21		

LN, lymph node.

**Table III:** Multivariate Cox regression survival analysis of clinicopathological parameters and S100A4.

Parameters	B	SE	Wald	Exp (B)	P-value
Age	0.072	0.034	4.467	0.930	0.035
Gender	0.350	0.361	0.937	1.419	0.333
Location	0.204	0.246	0.684	0.816	0.408
Size	1.474	0.471	9.799	4.369	0.002
Operation	0.221	0.245	0.816	1.248	0.366
Lauren	0.417	0.440	0.897	1.517	0.344
Histology	0.069	0.178	0.151	0.933	0.698
Differentiaiton	0.560	0.413	1.834	1.750	0.176
Borrmann	0.116	0.377	0.095	0.890	0.758
TNM	1.510	0.441	11.711	4.525	0.001
Metastasis	1.391	0.896	2.414	4.021	0.120
*H. pylori*	0.315	0.332	0.899	1.370	0.343
Expression	2.315	0.507	20.864	10.129	0.000

TNM, tumor-node-metastasis.
